# Letter regarding “Gastrointestinal foreign bodies in pet pigs: 17 cases”

**DOI:** 10.1111/jvim.16479

**Published:** 2022-07-08

**Authors:** Joe Smith, Paul Merkatoris, Ryan M. Breuer, Ricardo Videla, Pierre‐Yves Mulon, Jennifer Schleining, Dusty Nagy, Virginia Fajt, Shannon Reed, Jonathan P. Mochel, Amanda Kreuder, Patricia Dowling

**Affiliations:** ^1^ University of Tennessee Knoxville Tennessee USA; ^2^ Iowa State University Ames Iowa USA; ^3^ University of Wisconsin Madison Wisconsin USA; ^4^ Wisconsin Veterinary Diagnostic Laboratory Madison Wisconsin USA; ^5^ Texas A&M University College Station Texas USA; ^6^ College of Veterinary Medicine, Veterinary Diagnostic/Production Animal Medicine Iowa State University Ames Iowa USA; ^7^ Western College of Veterinary Medicine Saskatoon Saskatchewan Canada

Dear Editor,

We note the article, “*Gastrointestinal foreign bodies in pet pigs: 17 cases*” by Nakamae et al and applaud the authors for this contribution to the growing literature of miniature companion pig medicine. However, we have noticed an inconsistency in the language of the article, and discussion of such could serve to educate the community of veterinarians treating miniature companion pigs in North America.

The article states, “Authors declare no off‐label use of antimicrobials.” However, the authors report that the pigs in the study received multiple antimicrobials including “… cephalosporins (n = 8), beta‐lactams (n = 8), tetracyclines (n = 1), macrolides (n = 1), and aminoglycosides (n = 1).” The “no off‐label use” statement contradicts the reported administration of antimicrobial drugs, as none of these drugs are labeled for surgical prophylaxis or similar indications in the United States. Ceftiofur is labeled for the treatment of swine respiratory disease. While the specific beta‐lactams, tetracyclines, macrolides, and aminoglycosides administered are not specified in this report, the swine labelling of these drugs does not include surgical prophylaxis. Procaine penicillin G (beta‐lactam) is approved for pigs for treatment of erysipelas, oxytetracycline (tetracycline) is approved for treatment of bacterial pneumonia, tulathromycin (macrolide) is approved for the treatment of swine respiratory disease, and a formulation of gentamicin (aminoglycoside) exists for treatment of colibacillosis caused by *E. coli* in pigs up to 3 days of age. The article does not state if any of the pigs had concurrent conditions, specifically those the drugs are labeled for. Under the 2012 FDA Order of Prohibition regarding the use of cephalosporin drugs in major food‐producing animals (including swine as defined by 21 CFR 516.3^1^), the first specifically stated prohibition is for disease prevention purposes,^2^ and it should be noted that surgical prophylaxis could be classified as such.

All the aforementioned antimicrobial drugs can be used legally in an extra label manner in food animal species in the United States and Canada under a valid veterinary‐client‐patient relationship. This is an important distinction, as food animal status is determined by species, not role. While not common, there are documented cases of companion miniature pigs entering the human food chain.^3^ While this is an unlikely event, practitioners should receive and notify clients of withdrawal recommendations by contacting the Food Animal Residue Avoidance Databank (FARAD; US) or the Canadian global Food Animal Residue Avoidance Databank (CgFARAD) any time drugs are prescribed or administered to swine, regardless of the intended use of the animal.

We sincerely thank the authors' contribution to the growing body of literature about miniature pigs. In submitting this letter, we wish to emphasize the importance of adhering to extra label drug use requirements with respect to the current environment of antimicrobial stewardship, especially in relation to the potential dual consideration of miniature companion pigs as pet and food animals.

Respectfully,
